# Adherence to malaria treatment guidelines among health care workers in private health facilities in Kampala’s informal settlements, Uganda

**DOI:** 10.1371/journal.pgph.0002220

**Published:** 2023-09-05

**Authors:** Douglas Bulafu, Bridget Nagawa Tamale, Lesley Rose Ninsiima, James Natweta Baguma, Lydia Nabawanuka Namakula, Filimin Niyongabo, Grace Biyinzika Lubega, Dickson Aruhomukama, Rawlance Ndejjo, David Musoke

**Affiliations:** 1 Department of Disease Control and Environmental Health, School of Public Health, College of Health Sciences, Makerere University, Kampala, Uganda; 2 Department of Immunology and Molecular Biology, College of Health Sciences, School of Biomedical Sciences, Makerere University, Kampala, Uganda; ICMR-National Institute of Malaria Research: National Institute of Malaria Research, INDIA

## Abstract

Poor adherence to malaria treatment guidelines among healthcare workers (HCWs) is a major contribution to diagnostic challenges, treatment failure, and non-rational use of antimalarial medicines. However, there is limited information about adherence to malaria treatment guidelines among HCWs in private health facilities in informal settlements in Uganda. This study therefore assessed the level of adherence to malaria treatment guidelines and associated factors among HCWs in private health facilities in Kampala’s informal settlements. A cross-sectional study was conducted among 339 HCWs from private health facilities in slums of 4 selected divisions in Kampala, Uganda. Quantitative data was collected using a semi-structured questionnaire, cleaned in MS Excel 2016 and analyzed using STATA 15.0 statistical software. Bivariate and multivariate analysis were conducted using a generalized linear model of modified Poisson regression to obtain factors associated with adherence to malaria treatment guidelines. The study revealed that majority of respondents 71.1%(241/339) were aged 30 years and below, and 50.1%(170/339) of the were female. Almost all of the respondents 98.8%(335/339) reported that they had malaria diagnostic equipment (microscopy or rapid diagnostic tests) at their facilities, 47.5%(161/339) had non-recommended anti-malarial drugs present in stock and 36.0% reported that they did not refer severely ill patients to higher health facilities in the previous 3 months. Although 92.6%(314/339) of the respondents had heard about the national malaria treatment guidelines, 63.1%(214/339) of them adhered to these guidelines. Having a bachelors degree (APR 1.54, (CI: 1.13–2.10)P 0.006), and having high levels of knowledge (APR 1.44, (CI: 1.13–1.60)P 0.001) were positively associated with high adherence to malaria treatment guidelines. In conclusion, adherence to malaria treatment guidelines was suboptimal and less than the national target of 90%. Enforcement, supervision, trainings, and continuous medical education should be enhanced in private healthcare facilities to improve adherence to malaria treatment guidelines in informal settlements.

## Background

The global prevalence of malaria increased by 13 million cases from 2020 to 2022 [1]. Africa alone makes up 95% of such malaria cases and 96% of deaths (5% of deaths are from Uganda), with 80% of deaths among children under five years of age [2]. Although sub-Saharan African (SSA) countries are on track to reducing malaria incidence and mortality [3] due to preventive interventions such as the distribution of insecticide-treated bed nets (ITNs), indoor residual spraying (IRS), and Artemisinin-based Combination Therapy (ACT) [4], over half (55%) of the global malaria cases are from SSA [5].

The World Health Organization (WHO) introduced a global malaria strategy targeted to reduce malaria incidence and mortality rates by at least 90% by 2030 [6]. Guidelines preventing the sale of substandard drugs, misdiagnosis [7–10], wrong prescription of drugs [11] and non-rational use of antimalarial medicines by patients [10, 12, 13] were introduced to manage malaria treatment failure. These guidelines were adopted by the Government of Uganda (GOU) through the Ministry of Health National Malaria Control Program (NMCP) and other development partners. The GOU and partners are dedicated to controlling malaria, especially in slums with cost-effective, evidence-based prevention and treatment methods [14] to create a malaria-free country by 2030 [5].

Malaria, however, remains a major public health problem at both public and private health facilities in Uganda. The disease accounts for 30–50% of outpatient visits, 15–20% of all hospital admissions, and up to 20% of all hospital deaths [15]. In addition, a significant percentage of deaths due to malaria remain unknown and unregistered in the country [16]. The 2021 world malaria report ranked Uganda third with the highest number of malaria cases among malaria-endemic countries [5]. The rapid urbanization and the development of informal settlements (slums) (estimated at 20%) especially in Kampala [17–20], the country’s capital city, could be a contributing factor to high malaria incidence in Uganda. Slums have poor environmental health conditions [21, 22] that result in flooding and waterlogging increasing disease vectors, especially mosquitoes that transmit malaria [23, 24]. Several studies have revealed that slums in Kampala have a significantly high incidence of malaria, especially among children [25–27].

The majority of slums dwellers in Uganda obtain malaria treatment from private health facilities, which largely rely on using fever as a major symptom to diagnose malaria [28]. This contradicts the WHO malaria testing requirement of microscopy or rapid diagnostic test (RDT) and treatment with a quality-assured anti-malarial drug once confirmed positive [29]. Clinical diagnosis using fever creates a diagnostic dilemma, reduces the accuracy of this criterion for diagnosis of malaria since fever can occur in other illnesses as well [30, 31] and results in poor adherence to malaria treatment guidelines. Consequently, as the malaria burden increases, so does parasite resistance to treatment regimens [32, 33], and resource wastage [4], hence negative malaria treatment outcomes [34–39]. In addition, there is a dearth of information on adherence to malaria treatment guidelines among healthcare workers (HCWs) in private health facilities in informal settlements. Therefore, this study assessed the level of adherence to malaria treatment guidelines and associated factors among healthcare workers (HCWs) in private health facilities in Kampala’s informal settlements.

## Materials and methods

### Study design and setting

This was a cross-sectional study using quantitative data collection methods. The study was conducted in Kampala city, Uganda’s capital. Kampala has a population of over 3.6 million people, constituting 25% of the country’s total urban population [40, 41]. Administratively, the district is divided into 5 divisions (Central, Nakawa, Kawempe, Lubaga, and Makindye) each containing several slums where over 60% of the population lives [22]. These slums are characterized by poor solid waste management, excreta and wastewater management, drainage facilities, housing conditions, as well as poor vector and vermin control which are conducive breeding places for mosquitoes [21]. When infected, many slum dwellers acquire malaria treatment from private health facilities within these settings which are considered cheaper and easily accessible. These private facilities largely rely on fever as a major symptom to diagnose malaria [28].

### Study population and sampling

The study was conducted among HCWs in private health facilities in Kampala’s informal settlements. The sample size was determined using the formula by Kish Leslie considering a prevalence of 27.3% adherence to malarial treatment guidelines [8]. A sampling error of 5% and a non-response rate of 10% were considered giving a sample size of 339. Multi-stage sampling was employed in the study. Four divisions in Kampala with the highest slum population were purposively selected, and two slums in each division were randomly sampled. Mapping and listing of the private health facilities were conducted in the selected slums to obtain a list of private health facilities. The list of private healthcare facilities was inserted into a random sample generator to randomly obtain the health facilities that were included in the study. One health worker was randomly chosen per health facility to participate in the study.

### Data collection

Recruitment of participants and data collection was conducted from 26^th^ April– 20^th^ June 2022. Quantitative data were collected by trained research assistants, with a bachelors degree in any health related course, using a semi-structured questionnaire uploaded on the Kobo collect mobile application. The research assistants were trained on the study and use of data collection questionnaire. The questionnaire was designed and adopted from similar studies [42–44] and the WHO guidelines for malaria treatment [11]. It was divided into 4 sections, which included: social demographic characteristics; knowledge on anti-malaria drug resistance adherence to malaria treatment guidelines; and factors affecting adherence to malaria treatment guidelines. Before field data collection, pretesting was conducted in Katanga slum in Kampala city, which was not part of the selected slums for the study. This was done to validate and amend the tool where appropriate.

### Measurement of adherence

A set of 13 questions was designed to assess adherence to malaria treatment guidelines among respondents (S2 Text). These question were adopted from a similar study [44] and the WHO guidelines for malaria treatment [11]. For each question, respondents with a response “always” scored 1, while those with a response “sometimes /never” scored 0. A composite score was then generated based on the summation of all responses. The mean score was then used as a cut-off. Respondents who obtained a total score above the mean were considered to have good adherence, while those with a total score equal to or below the mean were considered to have poor adherence.

### Measurement of knowledge of malaria treatment guidelines

Knowledge of malaria treatment guidelines was assessed using 9 questions (S1 Text). Questions assessing knowledge on malaria were adopted the WHO guidelines for malaria treatment [11]. Respondents with a “yes” response received one score, and 0 if otherwise. A composite score was then computed based on the total scores of correct responses. The mean score was then used as a cut-off. Respondents who obtained a total score above the mean were considered to have good knowledge, while those with a total score equal to or below the mean were considered to have poor knowledge.

### Data management and analysis

The data was collected using Android-enabled mobile phones loaded with the Kobo collect application software. The data was synchronized onto the server daily and only the principal investigator and data manager had access to it. The collected data was cleaned in MS Excel 2016 and analyzed using STATA 15.0 statistical software. Descriptive analyses such as frequencies, proportions, and means (where appropriate) were performed for demographic characteristics, knowledge of antimalarial resistance, adherence to guidelines, and associated factors. To assess the association between the outcome variables (level of adherence) and explanatory variables, generalized linear model of modified Poisson regression was conducted providing Crude Prevalence Risk ratios (CPRs) and their corresponding 95% confidence intervals (CIs). Variables with p<0.05 were added to the multivariate analysis to ascertain significant variables for each outcome. The statistical significance levels were two-sided at p<0.05.

### Ethics approval and consent to participate

Ethical approval was obtained from the Makerere University School of Public Health Research and Ethics Committee (MakSPH-REC) (REC No: SPH-2021-207). The study was also registered by Uganda National Council for Science and Technology (REG No. HS2161ES). Administrative clearance was obtained from the Department of Public Health and Environment at Kampala City Council Authority. Study participants provided written informed consent at the initiation of data collection. The data collected was stored on drives that were only accessed by the principal investigator and data manager.

## Results

### Social demographics characteristics

Out of the 339 respondents, 50.1% (170/339) were females, more than two-thirds, 71.1% (241/339) were aged 30 years and below, and 51.9% (176/339) were enrolled nurses / midwives. In terms of education, 50.7% (172/339) of respondents were national certificate holders, The majority of respondents 68.4% (232/339) had five or fewer years of experience in the management and treatment of malaria cases, 74.3% (252/339) had an average monthly expenditure of 150 USD and below, and 57.8% (196/339) had not been supervised by authorities in the previous 6 months.

### Health facility characteristics

Considering the previous 3 months, almost all respondents reported having had malaria diagnostic equipment (microscopy or RDTs) (98.8% (335/339)) had recommended anti-malarial drugs 99.7% (338/339) at their health facility. Nearly half, 47.5% (161/339) respondents reported availability of non-recommended anti-malarial drugs in the previous 3 months (Table 1).

**Table 1:** Respondents’ socio-demographic and health facility characteristics.

Variable	Attribute	Frequency (n = 339)	Percentage (%)
**Healthcare worker characteristics**			
** Sex**	Male	169	49.9
	Female	170	50.1
**Age (years)**	≤30	241	71.1
	>30	98	28.9
** Highest Academic Qualification**	National certificate	172	50.7
	Diploma	128	37.8
	Bachelors degree	39	11.5
**Profession**	Nursing officer/assistant	36	10.6
	Clinical officer	70	20.6
	Laboratory technician/assistant	38	11.2
	Enrolled nurse/midwife	176	51.9
	Doctor	19	5.6
**Experience in the management and treatment of malaria cases (years)**	≤5	232	68.4
	>5	107	31.6
**Average monthly expenditure (US dollars)**	≤150	252	74.3
	>150	87	25.7
**Training in malaria case management and treatment in the previous 3 years**	No	127	37.5
	Yes	143	42.4
**Supervised in malaria case management and treatment in the previous 6 months**	No	196	57.8
	Yes	143	42.2
**Frequency of supervision (n = 143)**	Once	62	18.3
	Twice	42	12.4
	≥3	39	11.5
**Private Health Facility Characteristics**			
**Malaria diagnostic equipment (microscopy or RDTs) present in the previous 3 months**	No	4	1.2
	Yes	335	98.8
**Malaria diagnostic equipment was present in the previous 3 months**	Both microscopy and RDTs	152	44.8
	Microscopy	15	4.4
	RDTs	168	49.6
**Recommended anti-malarial drugs present in stock in the previous 3 months**	No	1	0.3
	Yes	338	99.7
**Recommended anti-malarial drugs present in stock in the previous 3 months (n = 338)**	Artemether-lumefantrin (AL)	326	96.2
	Artesunate-amodiaquine (AA)	283	83.5
	Quinine Injection	307	90.6
	Artemether Injection	264	77.9
	Sulphodoxinepyrimethamine (S-P)	338	99.7
**Non-recommended anti-malarial drugs present in stock in the previous 3 months**	No	178	52.5
	Yes	161	47.5
**Non- recommended anti-malarial drugs present (n = 161)**	Artesunate (monotherapy)	99	29.2
	Amodiaquine	20	5.9
	Chloroquine	16	4.7
	Halofantrine	9	2.7
	Dihydroartemisinin-piperaquine (DHA-PPQ)	111	32.7

### Factors influencing the type of anti-malarial drugs prescribed

Regarding factors influencing the type of anti-malarial drugs prescribed, more than three quarters of the respondents 80.2% (272/339) agreed to anti-malarial drug availability, 94.1% (319/339) agreed to anti-malarial drug effectiveness, 85.8% (291/339) agreed to existing national malaria treatment guidelines, 48.1% (163/339) agreed to drug promotion by manufacturers, 64.0% (217/339) agreed to patient preferences, and 57.8% (196/339) agreed to the need to make profits.

### Knowledge of the national guidelines for the diagnosis and treatment of malaria

Most respondents 92.6% (314/339) reported that they had heard about the national malaria treatment guidelines mostly through training and continuous medical education (70.2%). Almost all of the respondents 95.6% (324/339) agreed that malaria was clinically suspected mostly based on fever or a history of fever. More than three quarters of the respondents 78.8% (267/339) agreed that treatment solely based on clinical suspicion was only considered when a parasitological diagnosis was not accessible in a severely ill patient, and 86.1% (292/339) agreed that ACTs were the recommended treatments for uncomplicated falciparum malaria. The majority of respondents 95.9% (325/339) agreed that the first-line treatment for uncomplicated malaria was an ACT (Artemether/Lumefantrine—AL), 85.8% (291/339) agreed that the second-line treatment for uncomplicated malaria was an ACT (Dihydroartemisinin Piperaquine—DHA-PPQ), and 95.9% (325/339) agreed that Artesunate (given intravenously) was recommended for the treatment of severe malaria (Table 2).

**Table 2:** Knowledge of the national guidelines for the diagnosis and treatment of malaria.

Variable	Attribute	Frequency (N = 339)	Percentage (%)
**Heard about the National Malaria Diagnosis and Treatment Guidelines**	No	25	7.4
	Yes	314	92.6
Heard information from (n = 314)*	Colleagues	54	15.9
	Seminar/ clinical meetings	110	32.4
	Training/continuous medical education (CME)	238	70.2
	Internet	64	18.9
**Malaria is clinically suspected mostly based on fever or a history of fever and clinical suspicion of malaria should be confirmed with a parasitological diagnosis.**	No	15	4.4
	Yes	324	95.6
**Prompt parasitological confirmation by microscopy, or RDTs, is recommended in all patients suspected of malaria before treatment is started.**	No	6	1.8
	Yes	333	98.2
**Treatment solely based on clinical suspicion should only be considered when a parasitological diagnosis is not accessible in a severely ill patient**	No	72	21.2
	Yes	267	78.8
**ACTs are the recommended treatment for uncomplicated falciparum malaria.**	No	47	13.9
	Yes	292	86.1
**The artemisinin derivative components of the combination must be given for at least 3 (three) days for an optimum effect**	No	22	6.5
	Yes	317	93.5
**Anti-malarial treatment should be limited to test positive cases and negative cases should be reassessed for other common causes of fever.**	No	19	5.6
	Yes	320	94.4
**The first line treatment for uncomplicated malaria is an ACT, currently Artemether/Lumefantrine (AL).**	No	14	4.1
	Yes	325	95.9
**The second line treatment for uncomplicated malaria is also an ACT, Dihydroartemisinin Piperaquine (DHA-PPQ). Quinine will be the alternative second-line treatment**	No	48	14.2
	Yes	291	85.8
**Artesunate (given intravenously) is the recommended medicine for the treatment of severe malaria.**	No	14	4.1
	Yes	325	95.9

* Multiple choice question

### Adherence to the national guidelines for the diagnosis and treatment of malaria

Regarding diagnosis of malaria, over 95% of respondents reported always asking about symptoms of the illnesses 98.5% (334/339), presence or history of fever 96.5% (327/339), and signs of severe illness 93.5% (317/339). Most respondents 93.8% (318/339) always took both medical history and physical examinations appropriately, and 85.5% (290/339) always recommended/offered a blood test (RDT and microscopy) for malaria (even if the patient refused to test). Regarding treatment, more than three quarters of the respondents 86.7% (294/339) reported that they used Artesunate Quinine (given intravenously) to treat severe malaria, 73.7% (250/339) used Quinine treatment when ACTs were contraindicated, and 64.0% (217/339) referred severely ill patients (Table 3).

**Table 3:** Adherence to the national guidelines for the diagnosis and treatment of malaria.

Variable	Attribute	Frequency (N = 339)	Percent (%)
**Healthcare Worker (HCW) asked about the symptoms of the illness**	Sometimes/never	5	1.5
	Always	334	98.5
**HCW inquired if the patient has a fever/history of fever**	Sometimes/never	12	3.5
	Always	327	96.5
**HCW asked about any signs of severe illness (headache, convulsions, unable to eat or drink, vomiting)**	Sometimes/never	22	6.5
	Always	317	93.5
**HCW asked for the patient’s age**	Sometimes/never	6	1.8
	Always	333	98.2
**HCW weighed a patient using a weighing scale**	Sometimes/never	64	18.9
	Always	275	81.1
**HCW took body temperature with a thermometer**	Sometimes/never	15	4.4
	Always	324	95.6
**HCW took both medical history and physical examinations appropriately**	Sometimes/never	21	6.2
	Always	318	93.8
**HCW recommended/offered a blood test (RDT and microscopy) for malaria (even if the patient refuses the test)**	Sometimes/never	49	14.5
	Always	290	85.5
**HCW prescribed anti-malarial drugs for patients with positive RDT and microscopy results**	Sometimes/never	12	3.5
	Always	327	96.5
**HCW used ACTs to treat uncomplicated malaria**	Sometimes/never	30	8.8
	Always	309	91.2
**HCW used Artesunate Quinine (given intravenously) to treat severe malaria**	Sometimes/never	45	13.3
	Always	294	86.7
**HCW used Quinine treatment when ACTs are contraindicated**	Sometimes/never	89	26.3
	Always	250	73.7
**Referred severely ill patients to a higher health facility**	Sometimes/never	122	36.0
	Always	217	64.0

## Factors associated with adherence to malaria treatment guidelines

Less than two-thirds of the respondents 63.1% (214/339) had a high level of adherence to malaria treatment guidelines. At bivariate analysis, sex, age, highest level of education, cadre of health worker, level of knowledge were associated with adherence to malaria treatment guidelines. After adjusting for potential confounders having a bachelors degree as the highest level of education, being an enrolled nurse/midwife, and having high knowledge on malaria treatment guidelines were significantly associated with having good adherence to malaria treatment guidelines. Respondents who were female were 24% less likely to have a high adherence to malaria treatment guidelines as compared to males (APR = 0.76, 95% CI:0.63–0.91). Respondents who had a bachelors degree were 1.54 times more likely to have good adherence to malaria treatment guidelines as compared to those with certificates (APR = 1.54, 95% CI:1.13–2.10). Respondents who were enrolled nurses/midwives had a 51% higher likelihood of having good adherence to malaria treatment guidelines as compared to nursing assistants (APR = 1.51, 95% CI:1.00–2.27). Respondents who had high level of knowledge on malaria treatment guidelines were 1.35 times more likely to have good adherence to malaria treatment guidelines as compared to those who had low knowledge (APR = 1.35, 95% CI:1.13–1.60) (Table 4).

**Table 4:** Factors associated with adherence to malaria treatment guidelines among health care workers in private health facilities in informal settlements, Kampala, Uganda.

Variable	Attribute	Adherence to MTG		Crude PR (95% CI)	P-values	Adjusted PR (95% CI)	P-values
		Poor (n = 125)	Good (n = 214)				
**Sex of the HCW**	Male	47 (37.9)	122 (57.0)	1		1	
	Female	78 (62.4)	92 (43.0)	0.75 (0.63–0.89)	**0.001**	0.76 (0.63–0.91)	** 0.003**
**Age (years)**	≤ 30	99 (79.2)	142 (66.4)	1			
	˃ 30	26 (20.8)	72 (33.6)	1.25 (1.06–1.46)	** 0.007**	1.14 (0.97–1.34)	0.116
**Highest Academic Qualification**	Certificate	75 (60.0)	97 (45.3)	1	** **	1	
	Diploma	39 (31.2)	89 (41.6)	1.23 (1.03–1.47)	** 0.019**	1.22 (0.99–1.51)	0.057
	Bachelors degree	11 (8.8)	28 (13.1)	1.27 (1.00–1.61)	** 0.046**	1.54 (1.13–2.10)	** 0.006**
**Profession**	Nursing officer/Assistant	20 (16.0)	16 (7.5)	1		1	
	Clinical Officer	14 (11.2)	56 (26.2)	1.80 (1.22–2.64)	** 0.003**	1.29 (0.85–1.95)	0.224
	Laboratory technician/assistant	19 (15.2)	19 (8.9)	1.25 (0.69–1.83)	0.634	0.92 (0.56–1.49)	0.728
	Enrolled Nurse/midwife	65 (52.0)	111 (51.9)	1.42 (0.97–2.08)	0.073	1.51 (1.00–2.27)	** 0.048**
	Doctor	7 (5.6)	12 (5.6)	1.42 (0.86–2.35)	0.170	0.81 (0.46–1.42)	0.466
**Experience in the management and treatment of malaria**	≤5	91 (72.8)	141 (65.9)	1			
	˃ 5	34 (27.2)	73 (34.1)	1.22 (0.95–1.32)	0.172		
**Average monthly expenditure (USDs)**	≤150	95 (76.0)	157 (73.4)				
	> 150	30 (24.0)	57 (26.6)	1.05 (0.88–1.26)	0.585		
**Training in malaria case management and treatment in the previous 3 years**	No	45 (36.0)	82 (38.3)				
	Yes	80 (64.0)	132 (61.7)	0.96 (0.82–1.14)	0.669		
**Supervised in malaria case management and treatment in the previous 6 months**	No	79 (63.2)	117 (54.7)	1			
	Yes	46 (36.8)	97 (45.3)	1.14 (0.97–1.33)	0.121		
**Malaria diagnostic equipment (microscopy or RDTs) present in the previous 3 months**	No	3 (2.4)	1 (0.5)	1			
	Yes	122 (97.6)	213 (99.5)	2.54 (0.46–1.95)	0.282		
**Non recommended anti-malarial drugs present in stock in the last 3 months**	No	57 (45.6)	121 (56.5)	1			
	Yes	68 (54.4)	93 (43.5)	0.85 (0.72–1.00)	0.055		
**Heard about the National Malaria Diagnosis and Treatment Guidelines**	No	11 (8.8)	14 (6.5)	1			
	Yes	114 (91.2)	200 (93.5)	1.14 (0.79–1.63)	0.481		
**Level of knowledge on MTG**	Low	74 (59.2)	76 (35.5)	1			
	High	51 (40.8)	138 (64.5)	1.44 (1.20–1.72)	**<0.001**	1.35 (1.13–1.60)	** 0.001**
**Ever heard about anti-malarial drug resistance**	No	15 (12.0)	12 (5.6)				
	Yes	110 (88.0)	202 (94.4)	1.46 (0.95–2.24)	0.087		

## Discussion

This study assessed the level of adherence to malaria treatment guidelines and associated factors among health care workers in private health facilities in Kampala’s informal settlements. The knowledge of healthcare workers on malaria treatment guidelines was also investigated. Many health workers adhered to malaria treatment guidelines. High adherence was associated to having a bachelors degree as the highest level of education, being an enrolled nurse/midwife, and having high knowledge on malaria treatment guidelines. In addition, females were less likely to adhere to malaria treatment guidelines compared to their male counterparts. Nonetheless, some HCWs also had non recommended drugs in stock, did not refer patient to higher level facilities. These findings could be used by public health policymakers, administrators of private health facilities, local authorities, and other stakeholders to strengthen policies aimed at improving adherence to malaria treatment guidelines in private health facilities.

Although many health workers (63.1%) in this study adhered to malaria treatment guidelines, adherence levels were below the national malaria strategic plan target of 90% by 2020 [45]. The proportion of health workers who adhered to malaria treatment guidelines was also lower when compared to a study conducted in private clinics (85%) in Ethiopia [46]. The possible reasons for the lower levels of adherence could be attributed to various gaps in case management in private health facilities such as limited testing services [43]. On the contrary, adherence to malaria treatment guidelines was higher in our study when compared to another study conducted in private facilities in Ogun state in Nigeria (27.3%) yet it reported higher levels of knowledge [8]. This could imply that despite the healthcare workers having high levels of knowledge on malaria (as was the case in our study) which could improve adherence, there could be other health system (supplies) and patient-related factors affecting adherence to malaria treatment guidelines. For example, in this study, HCWs reported that availability of supplies such as antimalarial drugs (80.2%) and their effectiveness (94.1%) influenced the type of antimalarial treatment given to patients, which affecting adherence to guidelines despite their knowledge [8]. Therefore, private healthcare facility owners should ensure availability of adequate and effective antimalarials and equipment in their facilities to improve adherence to antimalarial guidelines among healthcare workers [8].

Our study revealed that healthcare workers with higher knowledge and education levels (bachelors degree and above) had high adherence to malaria treatment guidelines. This could be that their advanced training comes with better attention and improved clinical performance. Furthermore, in our study, HCWs who obtained information from training/ continuous medical education (70%) and seminars (32.4%) could have improved their knowledge levels. In addition, studies conducted in Uganda revealed that training health workers increased their adherence to treatment related to parasitological diagnosis with microscopy and RDTs from 24% in 2009 to 59% in 2013 [45]. Therefore, efforts towards training and continuous medical education of health workers at all levels, including the private sector, on the use of RDTs and microscopic diagnosis should be conducted to improve adherence to malaria treatment guidelines.

Almost half of the health facilities had non-recommended antimalarial drugs in the previous three months. Using non recommended anti-malarial increases the risk that patients will not receive the necessary dosage, which will lead to treatment failure, poor efficacy, and antimalarial drug resistance [45]. This also reflects an enforcement gap that needs to be addressed by the authorities.

In comparison to their male counterparts, this study revealed that female HCWs were less likely (APR 0.76, P-value 0.003), to adhere to malaria treatment guidelines. This could be attributed to many family work balance challenges, gender inequalities [47, 48], unequal pay, and limited opportunities for career development in health facilities [49] which potentially affects female health workers. In addition, a review conducted by Spoorthy MS et al., 2020 also showed that higher levels of despair, and discomfort was linked to being a woman with a middle-level professional position like being a health worker [50]. The review, however, was undertaken among studies conducted during COVID 19, the period in which this study was carried out. Therefore, the poor adherence could have been as a result of the effects of COVID 19. That notwithstanding, efforts aimed at improving working conditions for female HCWs should be enforced at workplaces including private health facilities.

This study revealed that a significant proportion (48.1%) of healthcare workers reported the demand from patients and manufacturers’ marketing influence as factors that influenced the types of drugs to be prescribed. Yet, evidence shows that drug promotion by manufacturers to providers is an important element that affects the drugs that HCWs prescription practices in private settings. This is concerning because it could lead to supplier-induced demand and superfluous prescriptions, aggravating the financial burden of the disease on the patients and also increasing the likelihood of antimalarial drug resistance. Furthermore, studies conducted in rural areas have revealed that non recommended antimalarial drugs pose an economic burden of US$2.6 million to the Ugandan government, and US$26.1 million in direct and indirect costs to patients due to loss of productivity and death [51]. These high costs are mostly imposed on the poor with a concentration index of 2.8 such as those living in slums [51]. Therefore, enforcement and supervision should be conducted in private healthcare facilities to ensure that only recommended drugs are in private health facilities [52].

A significant proportion of health workers in our study did not prescribe antimalarial drugs to malaria positive patients, and more than a third 36% of health facilities did not refer severely ill patients to higher health facilities in the previous 3 months. Non-referral to higher level health facilities could likely be attributed to HCWs perceptions that referral leads to loss of prestige, profit, as well as poor quality of care at private facilities [53]. In addition, patient factors such as high levels of poverty, and low levels of knowledge at the household level could have been attributed to the poor referral practices [53] as this study was conducted in slums. Therefore, sensitization and training on improving referral from private health facilities should be enforced to improve malaria treatment outcomes.

Our study had some limitations such as being prone to recall bias due to some questions asking for information of many months in the past. Furthermore, participants may have over-reported adherence as part of social desirability bias, because it was measured using self-reports. Despite these limitations, the study results are very important because they give a current assessment of the state of the adherence to malaria treatment guidelines in informal settlements in Kampala city.

## Conclusion

Adherence to malaria treatment guidelines was suboptimal and less than the national target of 90% adherence. Adherence was associated to having a bachelors degree as the highest level of education, being an enrolled nurse/midwife, and having high knowledge on malaria treatment guidelines. However, many health workers did not refer patients to higher facilities, and had in stock non-recommended antimalarial drugs. Therefore, routine supervision, training on malaria treatment guidelines, and continuous medical education should be conducted among healthcare workers to improve adherence to malaria treatment guidelines. In addition, the effectiveness of these interventions should also be studied further to establish their impact on the level of adherence to malaria treatment guidelines.

## Supporting information

S1 Text Knowledge on malaria treatment guidelines tool. (PDF)

S2 Text Adherence to malaria treatment guidelines tool. (PDF)

## List of abbreviations

MOH: Ministry of Health
MTG: Malaria Treatment Guidelines
WHO: World Health Organization
SDG: Sustainable Development Goals
ACT: Artemisinin-based Combination Therapy
DHA-PPQ: Dihydroartemisinin Piperaquine
