# Supplementary material for: Adherence to malaria treatment guidelines among health care workers in private health facilities in Kampala’s informal settlements, Uganda
Source: PLOS Glob Public Health. 2023 Sep 5;3(9):e0002220. doi: 10.1371/journal.pgph.0002220 (PMC10479897; doi:10.1371/journal.pgph.0002220)
Supplement: S1 Text — (PDF) [file pgph.0002220.s001.pdf]

Knowledge on malaria treatment guidelines (KMTG) tool

Knowledge on malaria treatment guidelines was assessed using 9 questions. Respondents with a "yes" response received one score, and 0 if otherwise. A composite score was then computed based on the total scores of correct responses. Respondents with a total score above the mean were considered as having high knowledge on malaria treatment guidelines. While, those with a total score equal to or below the mean were considered as having low knowledge.

**The statements below are related to the diagnosis and treatment of Malaria cases (Please Circle "Yes" if you agree or "No" if you do not agree with it or "Not sure")**		
**C4**	Malaria is clinically suspected mostly on the basis of fever or a history of fever and clinical suspicion of malaria should be confirmed with a parasitological diagnosis.	A. Yes<br>B. No<br>C. Not Sure
**C5**	Prompt parasitological confirmation by microscopy, or RDTs, is recommended in all patients suspected of malaria before treatment is started.	A. Yes<br>B. No<br>C. Not Sure
**C6**	Treatment solely on the basis of clinical suspicion should only be considered when a parasitological diagnosis is not accessible in a severely ill patient	A. Yes<br>B. No<br>C. Not Sure
**C7**	ACTs are the recommended treatments for uncomplicated falciparum malaria.	A. Yes<br>B. No<br>C. Not Sure
**C8**	The artemisinin derivative components of the combination must be given for at least 3 (three) days for an optimum effect	A. Yes<br>B. No<br>C. Not Sure
**C9**	Anti-malarial treatment should be limited to test positive cases and negative cases should be reassessed for other common causes of fever.	A. Yes<br>B. No<br>C. Not Sure
**C10**	The first line treatment for uncomplicated malaria is an ACT, currently Artemether/Lumefantrine (AL).	A. Yes<br>B. No<br>C. Not Sure
**C11**	The second line treatment for uncomplicated malaria is also an ACT, DihydroartemisininPiperaquine (DHA-PPQ). Quinine will be the alternative second line treatment	A. Yes<br>B. No<br>C. Not Sure
**C12**	Artesunate (given intravenously) is the recommended medicine for the treatment of severe malaria. Intravenous Quinine or Intramuscular Artemether are the alternatives to be used when Artesunate is not available.	A. Yes<br>B. No<br>C. Not Sure
