# Supplementary material for: Adherence to malaria treatment guidelines among health care workers in private health facilities in Kampala’s informal settlements, Uganda
Source: PLOS Glob Public Health. 2023 Sep 5;3(9):e0002220. doi: 10.1371/journal.pgph.0002220 (PMC10479897; doi:10.1371/journal.pgph.0002220)
Supplement: S2 Text — (PDF) [file pgph.0002220.s002.pdf]

Adherence to malaria treatment guidelines (AMTG) tool

A set of 13 questions was designed to assess adherence to malaria treatment guidelines among respondents. On each question, respondents with a response "always" scored 1, while those with sometime/never scored 0. A composite score was then generated based on the summation of all responses with all the correct responses "always". Then, the mean score was used as a cut off. Respondents who obtained a total score above the mean (11) were considered to have good adherence, while those with a total score equal to or below the mean where considered to have poor adherence.

**The statements below are related to the practices of HCWs in regards to the diagnosis and treatment of malaria cases. They will be used to measure the level of adherence to the guidelines. (Please circle either "Always" or "Sometimes" or "Never" accordingly)**		
**D1**	Healthcare Worker (HCW) asks about symptoms of the illness	A. Always<br>B. Sometimes<br>C. Never
**D2**	HCW inquires if the patient has fever/history of fever	A. Always<br>B. Sometimes<br>C. Never
**D3**	HCW asks about any signs of severe illness (headache, convulsions, unable to eat or drink, vomiting)	A. Always<br>B. Sometimes<br>C. Never
**D4**	HCW asks for patient's age	A. Always<br>B. Sometimes<br>C. Never
**D5**	HCW weighs a patient using a weighing scale	A. Always<br>B. Sometimes<br>C. Never
**D6**	HCW takes body temperature with a thermometer	A. Always<br>B. Sometimes<br>C. Never
**D7**	HCW takes both medical history and physical examinations appropriately	A. Always<br>B. Sometimes<br>C. Never
**D8**	HCW recommends/offers a blood test (RDT and microscopy) for malaria (even if patient refuses test)	A. Always<br>B. Sometimes<br>C. Never
**D9**	HCW prescribes anti-malarial drugs for patients with positive microscopy results	A. Always<br>B. Sometimes

		C. Never
**D10**	HCW prescribes anti-malarial drugs for patients with positive microscopy test results	A. Always<br>B. Sometimes<br>C. Never
**D11**	HCW uses ACTs to treat uncomplicated malaria	A. Always<br>B. Sometimes<br>C. Never
**D12**	HCW uses Artesunate Quinine (given intravenously) to treat severe malaria.	A. Always<br>B. Sometimes<br>C. Never
**D12**	HCW uses Quinine treatment when ACTs are contraindicated	A. Always<br>B. Sometimes<br>C. Never
**D13**	Do you refer severely ill patients	A. Always<br>B. Sometimes<br>C. Never
